# Fungous Growth of the Pulp

**Published:** 1900-11

**Authors:** Julio R. Martinez


### FUNGOUS GROWTH OF THE PULP.

BY JULIO R. MARTINEZ, D.D.S.

On December 12, 1898, Marion J., a mulatto woman, twenty years old, came under my care complaining of a very sore tooth,—the second right lower molar,—which presented a cavity of decay occupying the whole masticating surface and also portions of the buccal and labial surfaces. The bulk of this cavity was completely covered by an hypertrophied pulp, which had a highly congested appearance and was rather spheroidal in form. According to the patient, the growth had started two years ago, when by the action of decay the pulp was exposed. At first no appreciable soreness was felt, but later on it became quite troublesome, especially whenever food was introduced in the cavity or pressure was exerted during mastication.

As the only means to get rid of the growth, I proceeded at once to remove it. After isolating the tooth by the rubber dam, I applied some crystals of trichloracetic acid and allowed them to remain for five minutes. Then by means of a sharp spoon excavator, which had been sterilized, I removed the growth from its base. Then I wiped the cavity with phenol-sodique and applied a dressing of iodine to the remaining portions of the pulp, sealing the cavity with a temporary stopping of gutta-percha. Three days after I renewed the iodine dressing, and at the third sitting, a week after, I was able to remove the rest of the pulp almost painlessly. The canals were cleansed by means of sterilized broaches followed by injections of a three-per-cent. solution of hydrogen peroxide, and a temporary dressing of iodoform and oil of cloves was sealed in the canals. A week after, as the tooth seemed to be in a healthy condition and the dressing was clean, I filled the canals with oxychloride of zinc, after having washed them again with hydrogen peroxide, followed by absolute alcohol and warm air, in order to insure complete dryness; and three days later I placed a permanent filling of amalgam in the cavity.

I had the opportunity to see the patient four months after she was dismissed, and learned from her that she has not felt the least amount of pain or discomfort since the treatment was completed.

